# STA-TSN: Spatial-Temporal Attention Temporal Segment Network for action recognition in video

**DOI:** 10.1371/journal.pone.0265115

**Published:** 2022-03-17

**Authors:** Guoan Yang, Yong Yang, Zhengzhi Lu, Junjie Yang, Deyang Liu, Chuanbo Zhou, Zien Fan

**Affiliations:** School of Automation Science and Engineering, Xi’an Jiaotong University, Xi’an, Shaanxi, China; Hanyang University, KOREA, REPUBLIC OF

## Abstract

Most deep learning-based action recognition models focus only on short-term motions, so the model often causes misjudgments of actions that are combined by multiple processes, such as long jump, high jump, etc. The proposal of Temporal Segment Networks (TSN) enables the network to capture long-term information in the video, but ignores that some unrelated frames or areas in the video can also cause great interference to action recognition. To solve this problem, a soft attention mechanism is introduced in TSN and a Spatial-Temporal Attention Temporal Segment Networks (STA-TSN), which retains the ability to capture long-term information and enables the network to adaptively focus on key features in space and time, is proposed. First, a multi-scale spatial focus feature enhancement strategy is proposed to fuse original convolution features with multi-scale spatial focus features obtained through a soft attention mechanism with spatial pyramid pooling. Second, a deep learning-based key frames exploration module, which utilizes a soft attention mechanism based on Long-Short Term Memory (LSTM) to adaptively learn temporal attention weights, is designed. Third, a temporal-attention regularization is developed to guide our STA-TSN to better realize the exploration of key frames. Finally, the experimental results show that our proposed STA-TSN outperforms TSN in the four public datasets UCF101, HMDB51, JHMDB and THUMOS14, as well as achieves state-of-the-art results.

## Introduction

With the development of multimedia and the popularization of mobile devices, video is gradually becoming one of the most extensively used communication media [1]. This trend has also promoted the development of video understanding technology in computer vision. Human action recognition is one of the important branches of video understanding technology [2]. The task of human action recognition is to classify a video that is usually various types of human actions and is widely used in video retrieval, human-computer interaction [3], security monitoring and other fields [4]. Compared to pictures, videos contain more information, but camera actions, differences in the scale and posture of human actions, and mutation of illumination conditions in the video also greatly increase the difficulty of action recognition [5]. At the same time, a video often contains some background information and video frames that contribute less to action recognition. Useless information also affects the recognition process [6]. Therefore, the search for a method that can focus on human actions in both space and time has always been a hot issue in human action recognition.

At first, the researchers used hand-crafted feature-based methods for action recognition, relying mainly on low-level features of video frames, such as HOG [7], HOF [8], etc. These features can be used for highly discriminative actions. However, it is difficult for the classifier to use them to correctly classify some complex actions or actions with high similarity. Recently, the rapid development of deep learning has made it possible to extract deep features in video frames, and related methods mainly rely on Convolutional Neural Network (CNN) to extract deep features from RGB images and stacked optical flows in the video or utilize LSTM [9] to extract dynamic features from deep convolutional feature sequences of multiple consecutive frames. Among them, the Temporal Segment Network (TSN) proposed by Wang et al. [10] is a typical representative of a deep feature extraction method based on CNN, which has achieved good results in human action recognition. TSN divides a video into several segments, then extracts a frame from each segment for recognition using CNN, and finally fuses the recognition results of each segment by a consensus function to obtain the final recognition result. This structure enables the network to capture long-term information in the video and provides a significant improvement in the accuracy of action recognition. Convolutional Neural Networks (CNNs) used in TSN, such as ResNet [11], BN-Inception [12], etc., all use Global Average Pooling (GAP) to obtain a global feature representation. However, GAP makes the network to give the same degree of attention to the features in each spatial area. Meanwhile, the method of random frame extraction in each segment does not guarantee that the extracted frames contribute significantly to the action recognition. All these shortcomings can cause great interference to action recognition.

Based on the above analysis, we propose a Spatial-Temporal Attention Temporal Segment Networks (STA-TSN) on the basis of preserving the ability to capture long-term information, which enables the network to focus on human actions and realize key frames exploration. Specifically, our main contributions are as follows:

- We propose a multi-scale spatial focus features enhancement strategy, which changes the way of obtaining global features directly through GAP in traditional CNNs. First, we use a soft attention mechanism with Spatial Pyramid Pooling (SPP) to extract multi-scale spatially focused features from the convolutional feature maps. Then we fuse the original convolution feature maps with the multi-scale spatial focus feature maps. Finally, GAP is used to obtain a global feature representation of the augmented spatial focus features.
- We design a deep learning-based key frame exploration method in TSN. The LSTM is used to explore the temporal dynamic features among the global feature representations of the sampled frames in each segment. The model can then adaptively learn temporal attention weights in each segment from the temporal dynamic features by using a soft attention mechanism. Meanwhile, we design a temporal-attention regularization to guide our key frames exploration module to better explore key frames.
- To verify the effectiveness of our model, we conducte experiments on four public datasets: UCF101, HMDB51, JHMDB and THUMOS14. The experimental results show that our proposed STA-TSN has significant improvement in action recognition accuracy compared to TSN and reaches the state-of-the-art on the four datasets.

The remaining sections are organized as follows. The second section describes related works on action recognition. The third section introduces our proposed method in detail. In the fourth section, we analyze the effectiveness of our proposed model based on the experimental results. We summarize and expect our work in the fifth section.

## Related works

We classify action recognition methods into two categories based on the different ways of extracting video features: hand-crafted feature-based methods and deep learning model-based feature extraction methods. Table 1 is the summary of the related works.

**Table 1:** The summary of the related works.

**hand-crafted features-based methods**	**Features**
Menthods based on spatio-temporal interest points	Easy to be affected by noise, less robust.
improved dense trajectories (IDT)	Much better than above, but it only extracts low-level features.
**CNN-based methods**	**Features**
Two-Stream	Based on CNN to extract features and fuse the result both RGB and optical flow.
C3D	Add temporal dimension but the number of parameters is huge.
TSN	Based on Two-Stream, and it can capture the long-term information in the video.
**Attention-based methods**	**Features**
HM-RNN	Only based on the spatial attention.
RSTAN	Using LSTM to realize the spatial and temporal attention.
STAN	A spatial-temporal attention network across different modalities

### Hand-crafted feature based methods

Initially, the researchers performed action recognition by extracting low-level features from the video. These methods start by extracting spatio-temporal interest points from the video. Laptev et al. [13] extended 2D Harris corner detection to the spatio-temporal domain and proposed a 3D Harris spatio-temporal interest point detection that was applicable to the video. In addition, Gabor-based detection and 3D Hessian-based detection operators were also proposed [14]. After extracting spatio-temporal interest points, action features around the interest points are required to be extracted, such as HOG, HOF, HOG3D, 3D-SIFT, MBH, etc. Furthermore, some feature coding methods such as Bag-of-Words Model (BOW) [15] were usually used to describe the action features. The methods based on spatio-temporal interest points were less robust. To overcome this problem, researchers proposed action recognition methods based on trajectory technology [16]. The most representative methods were the Dense Trajectory (DT) [17] and the Improved Dense Trajectory (IDT) [18]. However, these methods are only limited to extracting the features of the middle and low layers in the video. In some complex categories, there are large differences between the low-level features, or some categories are so similar that the classifier cannot classify them correctly.

### Deep learning based methods

With the great success of CNNs in computer vision, some CNN-based action recognition models have been proposed. The two-stream CNN proposed by Simonyan et al. [19] used Spatial Stream ConvNet with RGB image input and Temporal Stream ConvNet with stacked optical flow input to classify them respectively, and finally the scores of the two networks were combined as the final recognition result. This method greatly improved the accuracy of action recognition. However, none of these methods could obtain the correlation between multiple consecutive frames. As a result, Donahue et al. [9] utilized LSTM to obtain the connection between multiple frames. Wang et al. [10] proposed temporal segment network which enabled the network to capture long-term information in the video. Feichtenhofer et al. [20] suggested that different segments in TSN should have different importance, so they assigned different weights to different segments. Moreover, Ji et al. [21] proposed to expand 2D-CNN to 3D-CNN for action recognition by adding a time dimension and Carreira et al. [22] proposed a new Two-Stream Inflated 3D ConvNet (I3D) to extract temporal and spatial features of the video. This method enabled end-to-end training of the network, but the amount of parameters was huge.

In recent years, some action recognition models with attention mechanisms have been proposed. Sharma et al. [23] proposed a soft attention mechanism based on LSTM, in which they used the output of the LSTM at the current moment, where the input was the convolutional features at the same moment that computed the spatial attention weights of the convolutional features at the next moment. Wang et al. [24] extended Recurrent Neural Network (RNN) to Hierarchical Multi-scale RNN(HM-RNN) and proposed a hard attention model based on Gumbel-softmax. Furthermore, Du et al. [25] used LSTM to realize a spatial-temporal attention module by utilizing convolutional feature maps at multiple time steps from two-stream CNN to automatically learn a spatial-temporal feature vector. Li et al. [26] devised a general attention neural cell and proposed a spatio-temporal attention network across different modalities.

In conclusion, the limitations of the related works are as follows. First, the hand-crafted feature based methods can only extract the low-level features and it is difficult to use them to classify the complex actions. Second, most CNN-based methods cannot capture long-term information in the videos. Finally, most models lack the ability to capture key features and frames. Unlike the past works, we retain the advantages of TSN and propose a STA-TSN. We design a new form of global feature representation by fusing the original convolution features with the multi-scale spatial focus features obtained through a soft attention mechanism, and finally the global feature representation of multi-scale spatial focus features enhancement is obtained through GAP. Besides, we design a key frames exploration module based on deep learning to enable the TSN to adaptively identify the key frames in each segment.

## Proposed model

In this section, we describe our Spatial-Temporal Attention Temporal Segment Networks (STA-TSN) in detail.

Specifically, TSN makes the model capable of incorporating long-range temporal information of videos by dividing the video into several segments and randomly sampling one frame from each segment. However, the sampling approach does not guarantee that each frame from the segment contributes to action recognition. Therefore, we propose an STA-TSN to solve this problem. First, we divide the video into *N* segments *S*_*N*_ as shown in Eq (1)

$$\begin{array}{c} \{S_{1},S_{2},\cdots ,S_{n},\cdots ,S_{N}\},n=1,2,\ldots ,\text{N} \end{array}$$ (1)

Next, unlike TSN, we utilize a global sampling to obtain *k* frames from each segment. ***T***_*nt*_ indicates the *t*-th frame of the *n*-th segments. All the frames from a video can be expressed in Eq (2).

$$\begin{array}{c} \left\{(T_{11},T_{12},\ldots ,T_{1k}),(T_{21},T_{22},\ldots ,T_{2k}),\ldots ,(T_{n1},T_{n2},\ldots ,T_{nt},\ldots ,T_{nk}),\ldots ,(T_{N1},T_{N2},\ldots ,T_{Nk})\right\} \end{array}$$ (2)

**Fig 1:**
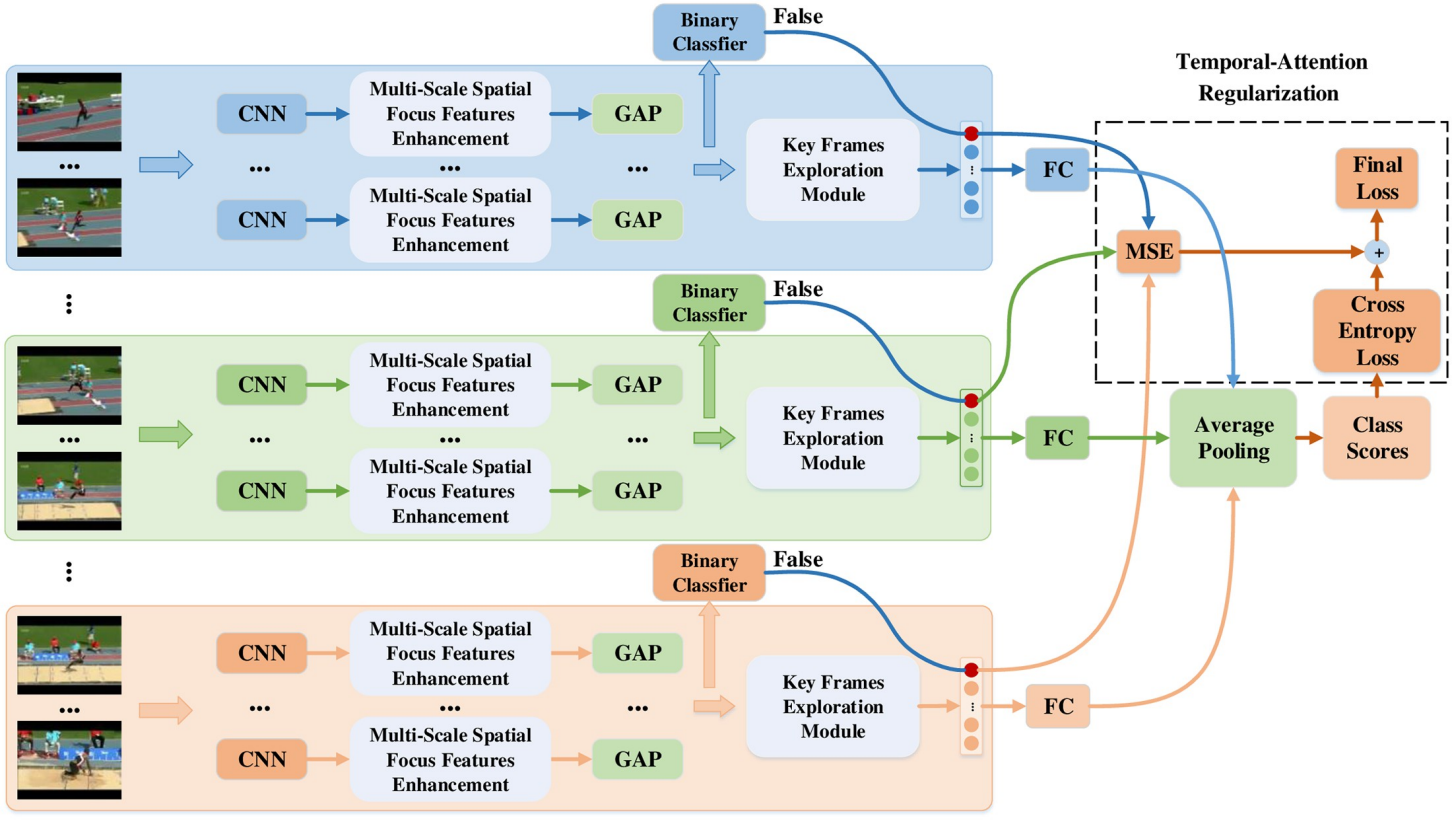
Video-level overview of our STA-TSN. The input video is divided into multiple segments (represented by different colors), and a Multi-Scale Spatial Focus Features Enhancement strategy is used to obtain the global feature presentment with spatial focus features enhancement. Then, the key frames exploration is realized using LSTM and a temporal-attention regularization is designed to guide our model to better explore the key frames. Eventually, the final class score is obtained by fusing the scores of all segments. Meanwhile, the same process is adopted for different modalities. Reprinted from [10] under a CC BY license, with permission from IEEE publisher, original copyright 2018.

Then, we design a CNN with multi-scale spatial attention to enhance spatial focus features based on the original features. Next, we design a key frames exploration module for the TSN to implement key frames exploration for each segment. Finally, a regularization is added to the cross-entropy loss function to guide the key frames exploration module to better explore key frames. The video-level model is shown in Fig 1, which will be explained next.

### Multi-scale spatial focus features enhancement

In a recent work [26], mentioning that using global features to predict actions could introduce noise from irrelevant regions, they designed a spatial attention cell and used spatial focus features to predict the action. However, it is difficult to guarantee that the spatial attention cell can capture all useful features. Therefore, we propose a compromise multi-scale spatial focus features enhancement strategy to strengthen the spatial focus features based on the original features. Meanwhile, to make the spatial attention cell get more informative, we use a SPP layer in an attempt to explore multi-scale spatial focus features.

**Fig 2:**
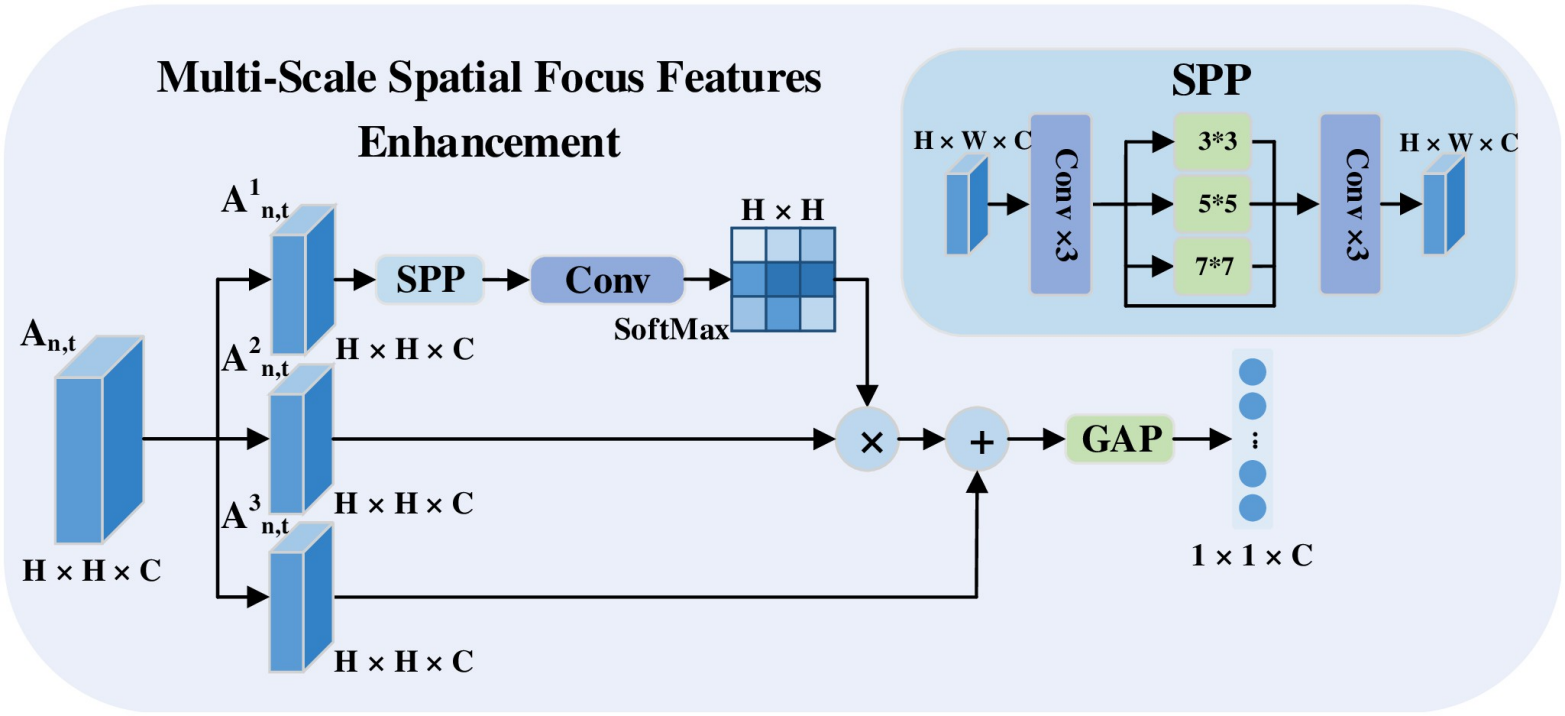
Details of our multi-scale spatial focus features enhancement strategy. The input of the module is the output of the last convolutional layer. First, a soft attention mechanism with SPP is used to obtain the multi-scale spatial features. Then, the spatial focus features are summed with the original features, and GAP is used to obtain the global feature representations with multi-scale spatial focus features enhancement.

As shown in Fig 2, for the *t*-th frame of the *n*-th segment, we obtain the feature map ***A***_*n*,*t*_ with a dimension of *H* × *H* × *C* from the last convolutional layer of CNN, where *H* × *H* is the number of pixels in a feature map and *C* is the dimension of the feature map (in our experiments, *H* = 7 and *C* = 2048). For a more effective depiction, we made three copies of ***A***_*n*,*t*_ and named them $A_{n,t}^{1}$, $A_{n,t}^{2}$, $A_{n,t}^{3}$.

For $A_{n,t}^{1}$ we use it to obtain the multi-scale spatial attention weights. The specific process is to first let $A_{n,t}^{1}$ pass through a SPP [27] with sizes of 1 × 1, 3 × 3, 5 × 5 and 7 × 7 which can pool features extracted at various scales and improve the robustness of the model, and then we adopt a soft attention mechanism to produce the attention vector from the multi-scale spatial features by using a convolutional layer with a 1 × 1 kernel activated by the softmax function. As shown in Eqs (3)–(5).

$$\begin{array}{c} \alpha_{n,t}=\{\alpha_{n,t}^{1,1},\cdots ,\alpha_{n,t}^{i,j},\cdots ,\alpha_{n,t}^{H,H}\} \end{array}$$ (3)

$$\begin{array}{c} \alpha_{n,t}^{i,j}=\frac{\text{exp}\;({\tilde{\alpha }}_{n,t}^{i,j})}{\sum_{m=1}^{H}\sum_{p=1}^{H}\text{exp}\;({\tilde{\alpha }}_{n,t}^{m,p})}, \end{array}$$ (4)

$$\begin{array}{c} {\tilde{\alpha }}_{n,t}=W^{T}(SPP(A_{n,t}^{1}))+b, \end{array}$$ (5)

where *W*, *b*, and ${\tilde{\alpha }}_{n,t}$ are the weights, bias and output of the 1 × 1 convolutional kernel, *SPP*(⋅) represents the output of the SPP layer and ***α***_*n*,*t*_ is the attention weight corresponding to the position whose coordinate is (*i*, *j*) on the feature map. Next, we obtain the multi-scale spatial focus features $f_{n,t}^{SF}$ by performing an inner product between ***α***_*n*,*t*_ and $A_{n,t}^{2}$ which is represented in Eq (6).

$$\begin{array}{c} f_{n,t}^{SF}=\alpha_{n,t}\cdot A_{n,t}^{2},n=1,2,\ldots ,N;t=1,2,\ldots ,k \end{array}$$ (6)

Finally, we add $f_{n,t}^{SF}$ and the copy of original features $A_{n,t}^{3}$ to enhance the spatially focused features based on the original features and then use a GAP to obtain the global feature representations ***F***_*n*,*t*_ with multi-scale spatial focus features enhancement, it is expressed in Eq (7).

$$\begin{array}{c} F_{n,t}=GAP(f_{n,t}^{SF}+A_{n,t}^{3}),n=1,2,\ldots ,N;t=1,2,\ldots ,k \end{array}$$ (7)

### Key frames exploration in TSN

During the sampling process, TSN randomly samples one frame from each segment as the representation of the whole segment. However, using this strategy may extract frames that are unrelated to the action. Inspired by the work of Zhu [28], which presented a deep learning approach to identify key volumes, we follow the elegant idea and design an LSTM-based key frames exploration module in the TSN to explore the key frames. Next, we will introduce the implementation process in detail.

**Fig 3:**
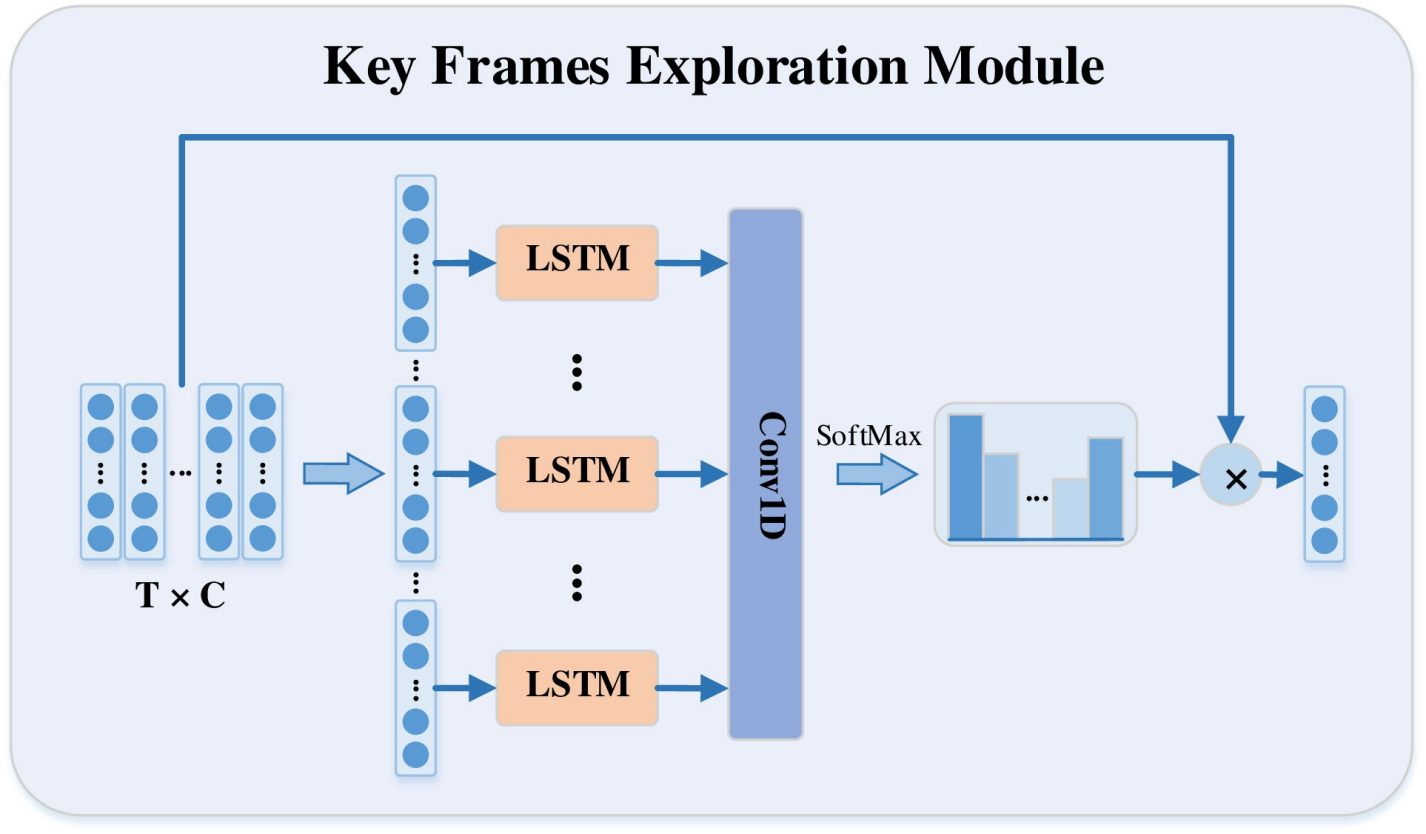
The details of key frames exploration. The input of the module is the global feature representations of the frames sampled from each segment. First, the LSTM is used to obtain the temporal dynamic features. Then, the temporal attention weights are obtained using the soft attention mechanism. Finally, the final segment feature representation is calculated by temporal attention weighting.

It is well known that LSTM [29] has an excellent ability to explore temporal dynamic features. However, most methods often use temporal dynamic features directly to predict the action. In contrast to these methods, an attempt was made to explore temporal attention using the temporal dynamic features obtained by the LSTM. Specifically, for example in the *n*-th segment, we first adopt the global feature representations ***F***_*n*,1_, ***F***_*n*,2_, ⋯, ***F***_*n*,*k*_ of the frames sampled from each segment as the input to the LSTM at each time step, as shown in Fig 3. Then, as shown in Eqs (9) and (10), with the output sequence ***h***_*n*,1_, ***h***_*n*,2_, ⋯, ***h***_*n*,*k*_ of all time steps of the LSTM, we use a convolutional layer with a 1 × 1 1-D kernel activated by the softmax function to produce the temporal attention vector ***β***_*n*_ indicated in Eq (8).

$$\begin{array}{c} \beta_{n}=\{\beta_{n,1},\beta_{n,2},\cdots ,\beta_{n,t},\cdots ,\beta_{n,k}\}, \end{array}$$ (8)

$$\begin{array}{c} \beta_{n,t}=\frac{\text{exp}\;({\tilde{\beta }}_{n,t})}{\sum_{m=1}^{k}\text{exp}\;({\tilde{\beta }}_{n,m})},n=1,2,\ldots ,N;t=1,2,\ldots ,k \end{array}$$ (9)

$$\begin{array}{c} {\tilde{\beta }}_{n,t}=W^{T}h_{n,t}+b. \end{array}$$ (10)

where *W*, *b*, and ${\tilde{\beta }}_{n,t}$ are the weights, bias and output of the 1 × 1 1-D convolutional kernel. Obviously, the value of ***β***_*n*,*t*_ indicates the degree of contribution of the *t*-th frame in the corresponding segment. Next, we fuse the feature representations of all sampled frames in each segment by temporal attention weighting as the final feature representation ***F***_*n*_ as shown in Eq (11).

$$\begin{array}{c} F_{n}=\sum_{t=1}^{k}\beta_{n,t}F_{n,k}. \end{array}$$ (11)

Then a fully connected layer is utilized as classifier to produce the class scores for all classes [30]. Meanwhile, the LSTM, convolutional layer, and fully connected layer share the parameters in all segments. Finally, the class scores of all segments are aggregated by average pooling to obtain the final class scores.

### Temporal-attention regularization

In the meantime, in order to guide our key frames exploration module to better explore the key frames, we design a temporal-attention regularization for the cross entropy loss function ***l***_*CE*_. However, the main problem is that we do not know in advance which frame is the key frame. Fortunately, since during training, we first train the CNN using a two-stream structure and then train the key frames exploration module. Hence, as shown in Fig 1, we can use features from the pre-trained CNN to train binary classifiers, where the number of binary classifiers is the same as the number of categories, to determine whether the current feature is the corresponding category. Based on these, we calculate the mean square error ***l***_*MSE*_ between the temporal attention weights ***β***_*n*,*t*_ corresponding to the frame and zero when the binary classifier judges the frame to be false in Eqs (12) and (13). With ***l***_*MSE*_, the final loss is written as Eq (14).

$$\begin{array}{c} l_{MSE}=\frac{1}{M}\sum_{n=1}^{N}\sum_{t=1}^{k}{\left({\hat{\beta }}_{n,t}\right)}^{2} \end{array}$$ (12)

$$\begin{array}{c} {\hat{\beta }}_{n,t}=\{\begin{array}{cc} \beta_{n,t}, & y=\;\text{false}\;\\ 0, & y=\;\text{true}\; \end{array} \end{array}$$ (13)

$$\begin{array}{c} l_{\text{final}\;}=l_{CE}+l_{MSE} \end{array}$$ (14)

where *M* is the number of frames that are judged to be false, and *y* is the result of the binary classifier. ***β***_*n*,*m*_ is the temporal attention weight of the *t*-th frame in the *n*-th segment and ${\hat{\beta }}_{n,m}$ is a function of ***β***_*n*,*m*_. Based on the temporal-attention regularization, our key frames exploration module can explore key frames more purposefully.

## Experiments

In this section, we evaluate our STA-TSN on four public datasets. First, we introduce the four public datasets. Then we describe the approach of implementation in detail. Next, we compare our STA-TSN with the baseline and other start-of-the-art techniques. Finally, to visually demonstrate the effectiveness of our STA-TSN, we randomly select several videos as samples to visualize spatial and temporal attention.

### Datasets

We mainly use four public datasets named UCF101 [31], HMDB51 [32], JHMDB [33] and THUMOS14 [34] for human action recognition. Especially, THUMOS14 is an untrimmed dataset, in which videos contain many irrelevant frames, we use it to further verify the effectiveness of the key frames exploration module.

UCF101 is a dataset of 101 human action classes from videos in the wild and consists of 13320 videos that contain 101 action classes, including five types: Human-Object Interaction, Body-Motion Only, Human-Human Interaction, Playing Musical Instruments and Sports. The dataset collectors provide three predefined train/test splits and we report the accuracy over the three splits.

HMDB51 is a large video database of 51 human motion classes, which captures richer and more complex human actions, contains 6766 videos with 51 action classes. It has five main types: Facial Expression, such as smiling, Facial Expressions-Object Interaction, such as smoking, Human-Object Interaction, such as horse riding, Body-Motion Only, such as climbing, Human-Human Interaction, such as hugging. It also has three predefined train/test splits and each split includes 3570 training and 1530 test videos. We evaluate the accuracy over the three splits.

The full name of JHMDB is Joint-annotated Human Motion Database. It is a fully annotated dataset for human actions and human poses and contains 928 videos with 21 action classes. It removes some of the videos and categories in HMDB51 where the action is not obvious. We also use the three train/test splits provided by the collectors to prove the validity of our model.

THUMOS14 is the public dataset of THUMOS Challenge 2014 which contains the same 101 categories as UCF101. It contains three parts, including training data, validation data and test data. The training data is the UCF101 dataset which includes 13320 trimmed videos. The validation data includes 1010 untrimmed videos and each includes one or multiple actions. The test data contains 1574 untrimmed videos and we report the result of the test data to validate our model. For this dataset, we use the official evaluation index, mean Average Precision (mAP) to evaluate our model. The detailed calculation formula is as Eqs (15)–(18).

$$\begin{array}{c} AP\left(i\right)=\frac{\sum_{v=1}^{V}\left(P\left(v\right)\times \text{rel}\left(v\right)\right)}{\sum_{v=1}^{V}\text{rel}\left(v\right)}, \end{array}$$ (15)

$$\begin{array}{c} P\left(v\right)=\frac{TP_{i}}{FP_{i}+TP_{i}}, \end{array}$$ (16)

$$\begin{array}{c} \text{rel}\left(v\right)=\{\begin{array}{c} 0,\;\text{prediction}\;\text{is}\;\text{false}\;\\ 1,\;\text{prediction}\;\text{is}\;\text{true}\;, \end{array} \end{array}$$ (17)

$$\begin{array}{c} mAP=\frac{1}{I}\sum_{i=1}^{I}AP\left(i\right), \end{array}$$ (18)

where *V* is the number of videos in the test data and the data is sorted in descending order by the final score. *TP*_*i*_ represents that the number of true positive up to *v*-th video for the *i*-th category and *FP*_*i*_ is the number of false positive up to *v*-th video for the *i*-th category. *I* is the number of class.

### Implementation details

The proposed STA-TSN is an end-to-end structure and we perform the structure using the following details.

In our experiment, the videos are first divided into three segments and 10 frames are globally sampled from each segment. For the network architecture, in general, a two-stream structure remains in use, which fuses a class score both in the spatial stream network with the input as RGB images and in the temporal stream network with the input as stacked 10 consecutive optical flows. Specifically, the optical flows are extracted by the TVL1 optical flow algorithm and rescale linearly in the range [0, 255]. ResNet-152, pre-trained on the ImageNet dataset [35], is used for convolutional feature maps in both spatial and temporal streams. In addition, the dimension of the hidden layer in the LSTM is 2048.

#### Network training

A two-stream structure is first used to train our ResNet152 with spatial attention (SA-ResNet152) via transfer learning [36]. Next, the weights of the key frames exploration module are trained by stochastic gradient descent, where the momentum is 0.9 and the mini-batch size is set to 128. The learning rate is initialized to 0.001 and decreased to its $\frac{1}{10}$ every 3000 iterations. The whole training process stops at 15000 iterations. We implement our architecture on PyTorch [37] and multi-GPUs parallel computing.

### Comparison with baselines

In this part, we set up two sets of comparative experiments to verify the effectiveness of our proposed structure. The entire comparative experiments are tested on the first dataset split of UCF101, HMDB51 and JHMDB. And for THUMOS14, we only test it on the second experiment. For a fair comparison, each model is tested with 30 frames extracted from the video using global sampling. For the each video, the final class scores are obtained by averaging the scores across the 10 crops of sampling frames, which can be obtained by cropping and flipping the center and four corners of the frame. Finally, we use the accuracy which can calculate by Eq (19) to evaluate the performance of the models.

$$\begin{array}{c} \;\text{Accuracy}\;=\frac{\sum_{i=1}^{I}TP_{i}}{\sum_{i=1}^{I}TP_{i}+\sum_{i=1}^{I}FP_{i}}, \end{array}$$ (19)

where *TP*_*i*_ and *FP*_*i*_ respectively indicate the number of correct and wrong predictions in the *i*-th class. *I* is the number of class.

- Under the original two-stream ConvNets (RGB + Flow) architecture, we choose ResNet152 as the baseline and compare our proposed SA-ResNet152 with it. As shown in Table 2, our SA-ResNet152 consistently outperforms the baseline on all three datasets. In particular, when the input is RGB, our SA-ResNet152 shows a significant improvement over the baseline on all three datasets. The improvement over the baseline is 0.5% for UCF101, 0.9% for HMDB51, and 1.5% for JHMDB. This is due to the fact that the attention structure in our module can adaptively extract focus features from original convolutional features. As shown in the section: spatial-temporal attention visualization, we superimpose the original image with the upsampled spatial atttention mask in the second line of each dashed box. It can be clearly seen that our module can accurately locate the spatial focus area in the image. At the same time, we fuse the extracted focus features with the original features, which further strengthens the weight of the focus features. Therefore, it can prove that our multi-scale spatial focus features enhancement strategy can use features more effectively for action recognition. When the optical flow is used as input, our SA-ResNet152 is basically equal to the baseline. The reason is that the extracted optical flow mainly captures human action information and filters out a lot of redundant information, resulting in no significant improvement in baseline for our SA-ResNet152.
- Under the original TSN architecture, we choose SA-ResNet152 as the baseline and compared it with our STA-TSN. In Table 2, our STA-TSN shows a significant improvement over the baseline on the four datasets. The improvement over the baseline is 0.7% for UCF101, 1.4% for HMDB51, and 3% for JHMDB. In particular, on the unedited dataset THUMOS14, our STA-TSN improves the accuracy and mAP of the baseline by 1.8% and 4.9%. This is because our STA-TSN effectively utilizes the temporal dynamic features extracted by the LSTM, which weakens the influence of unimportant frames on action recognition by assigning different weights to the convolutional features of different frames. It also proves that only spatial attention is not enough, and temporal attention is also very important in action recognition.

**Table 2:** Performances of the baseline and our proposed method on UCF101 (split1), HMDB51 (split1), and JHMDB (split1).

Model	UCF101 (split1)			HMDB51 (split1)			JHMDB (split1)			THUMOS14	
	RGB	Flow	Two	RGB	Flow	Two	RGB	Flow	Two	Two	
										Accuracy	mAP
ResNet152	82.3	84.2	91.6	51.5	57.2	67.5	54.5	64.2	72.8	-	-
**SA-ResNet152**	82.8	84.5	**91.9**	52.4	56.7	**67.6**	56.0	64.6	**73.2**	-	-
SA-ResNet152+TSN	82.7	87.6	92.1	50.1	59.5	67.2	56.7	65.3	78.0	56.8	63.5
**STA-TSN(ResNet152)**	83.4	86.5	**92.8**	53.9	56.6	**68.6**	58.6	69.8	**81.0**	**58.6**	**68.4**

**Fig 4:**
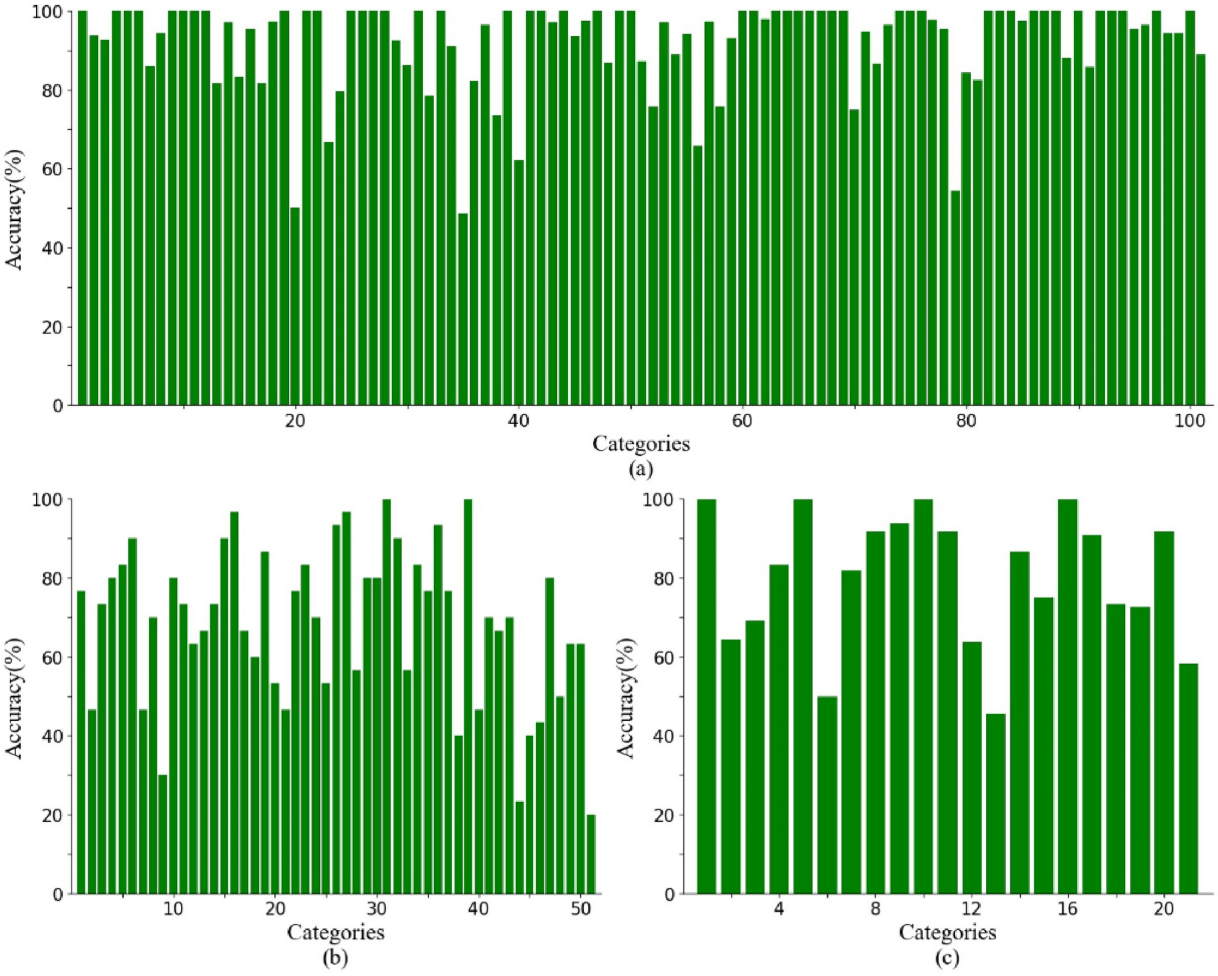
Category accuracy of the test set on three datasets (split 1) using our STA-TSN. (a) UCF101 dataset, (b) HMDB51 dataset, and (c) JHMDB dataset. Horizontal axis represents classes and the vertical axis shows accuracies for the corresponding class for the test set.

**Fig 5:**
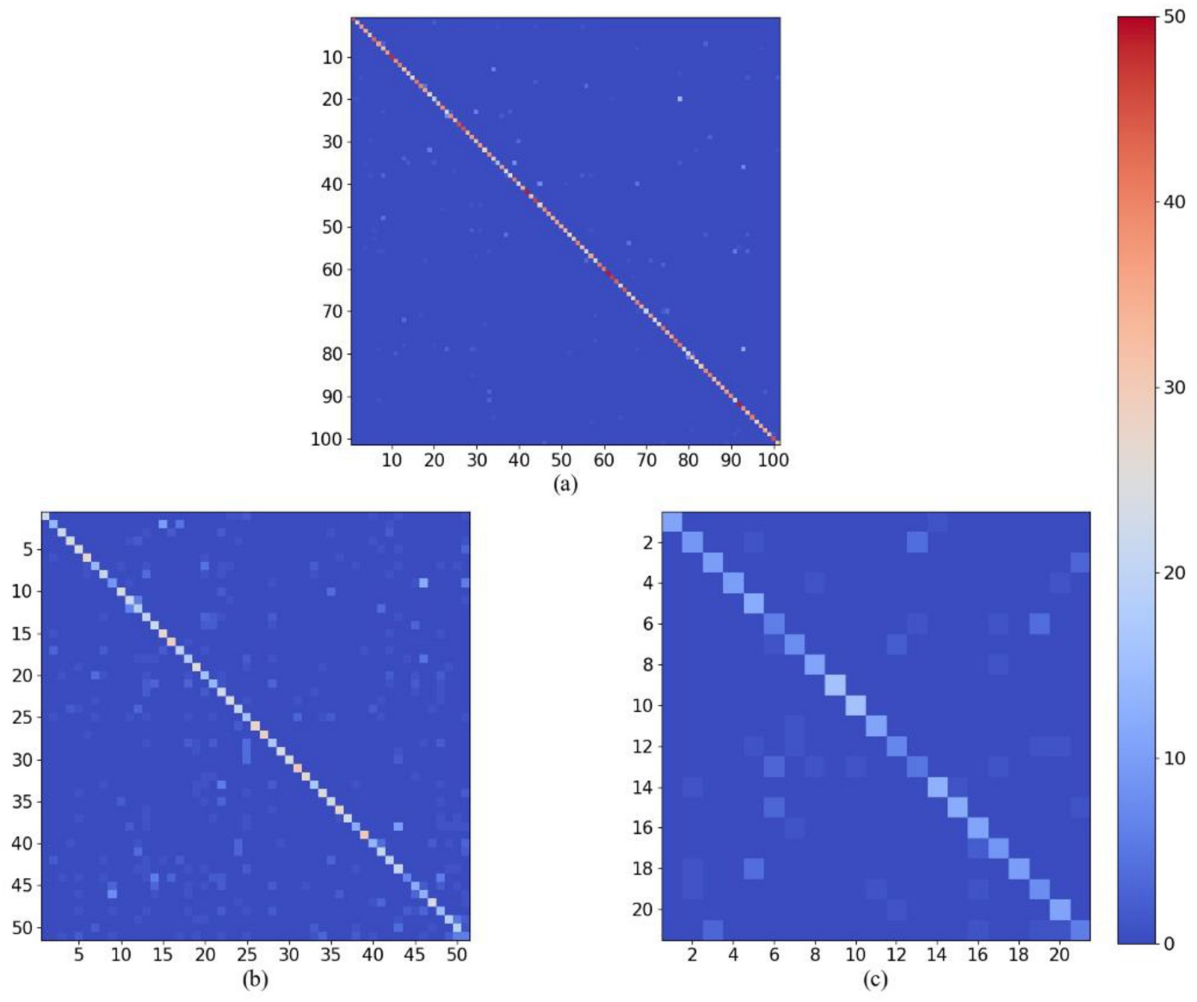
Confusion matrices for the three datasets using our STA-TSN. (a) UCF101 dataset, (b) HMDB51 dataset, and (c) JHMDB dataset. Horizontal axis represents predicted class, the vertical axis represents actual class and the main diagonal represents the true positives. The main diagonal is brighter, the number of the true positives is more.

In Fig 4, we visualize the accuracy of each category of our STA-TSN on the first split of the three datasets. For UCF101, the accuracies are above 85% for most classes and even approach 100% for some categories. There are only few class accuracies under 70%. From Fig 4(b), the most of classes are above 60% and the accuracy for all classes are above 40% except drawing sword, swinging baseball and waving. For JHMDB, all categories are above 50% except pushuping and swinging baseball. Meanwhile, we calculate the confusion matrix of our STA-TSN on the three datasets, as shown in Fig 5. From the picture, the true positive intensities are brighter for all most classes in three datasets.

### Comparison with state-of-the-art

In this part, we compare our STA-TSN with the state-of-the-art on UCF101, HMDB51 and JHMDB. For a fair comparison with other methods, as shown in Table 3, we conduct experiments on three different splits of UCF101, HMDB51 and JHMDB and average the accuracy of the three splits.

**Table 3:** Performances of our STA-TSN on UCF101 (all three splits), HMDB51 (all three splits), and JHMDB (all three splits).

	Split 1	Split 2	Split 3	Mean
**RGB-Stream**				
UCF101	83.4	81.5	81.2	82.1
HMDB51	53.9	49.8	49.3	51.0
JHMDB	58.6	55.2	54.3	56.0
**Flow-Stream**				
UCF101	86.5	89.8	89.7	88.7
HMDB51	56.6	58.2	59.8	58.2
JHMDB	69.8	66.3	67.6	67.9
**Two-Stream**				
UCF101	92.8	92.9	94.2	93.3
HMDB51	68.6	67.2	68.0	67.9
JHMDB	81.0	77.1	77.0	78.4

As shown in Table 4, we compare our STA-TSN with CNN-based approaches such as Two-Stream [19], C3D+iDT [38], Siamese network [39], Two-Stream Fusion [20] and LSTM-based approaches such as Composite LSTM [40], LRCN [9], VideoLSTM [41], LTC Network [42] and attention-based approaches such as AdaScan [43], Key Volume Mining [28], Hierarchical Attention Networks [24], RSTAN [25], STAN [26] on UCF101. The results indicate that our model reaches the state-of-the-art and even outperforms all the compared methods after two-stream fusion. Specifically, compared with CNN-based methods, our STA-TSN has both spatial and temporal attention so that our STA-TSN far exceeds CNN-based methods in both RGB and optical flow. Although LSTM-based approaches use LSTM to extract temporal dynamic features, they do not reprocess temporal dynamic features and directly use these features for classification. The result of LSTM-based approaches surpass CNN-based methods, there is still a certain gap compared with ours. Compared with attention-based approaches, our STA-TSN performs better after two-stream fusion, which also confirms the superiority of our method.

**Table 4:** Comparison with the state-of-the-art on UCF101 (average over three splits).

State-of-the-art	RGB	Flow	Two
Two-Stream [19]	-	-	88.0
C3D+iDT [38]	-	-	90.4
Siamese network [39]	80.8	87.8	92.4
Composite LSTM [40]	75.8	77.7	84.3
LRCN [9]	77.1	77.0	82.9
VideoLSTM [41]	79.6	82.1	88.9
LTC Network [42]	82.4	85.2	91.7
AdaScan [43]	78.6	83.4	89.4
Two-Stream Fusion [20]	-	-	92.5
ActionVLAD [44]	-	-	92.7
Key Volume Mining [28]	-	-	93.1
Hierarchical Attention Networks [24]	75.1	85.4	92.7
RSTAN [25]	-	-	92.5
STAN [26]	82.8	88.2	92.8
**STA-TSN**	82.1	88.7	**93.3**

The comparison results on HMDB51 are shown in Table 5, where our STA-TSN improves by 0.4% over the best competitor. And we get the same results as UCF101 which is that out STA-TSN outperforms CNN-based approaches, LSTM-based approaches, and attention-based approaches. For the JHMDB in Table 6, since most of the state-of-the-art methods on the JHMDB are based on pose-estimation, we only compare the final accuracy with the state-of-the-art methods. The results again demonstrate that our STA-TSN achieves the best performance. Meanwhile, the above method based on pose estimation needs to mark the bones of the people in the video, which greatly increases the extra works and costs. We directly use video framesas the input of the model, but we get better performance.

**Table 5:** Comparison with the state-of-the-art on HMDB51 (average over three splits).

State-of-the-art	RGB	Flow	Two
Two-Stream [19]	-	-	59.4
Siamese network [39]	44.1	57.1	62.0
VideoLSTM [41]	43.3	52.6	56.4
LTC Network [42]	49.7	59.0	64.8
AdaScan [43]	41.4	49.2	54.9
Two-Stream Fusion [20]	-	-	65.4
ActionVLAD [44]	51.2	58.4	66.9
Key Volume Mining [28]	-	-	63.3
Hierarchical Attention Networks [24]	47.7	58.3	64.3
Temporal-Inception [45]	-	-	67.5
**STA-TSN**	51.0	58.2	**67.9**

**Table 6:** Comparison with the state-of-the-art on JHMDB (average over three splits).

State-of-the-art	Accuracy
Two-Stream LSTM [46]	69.0
GRP+IDT+FV [47]	70.6
RSTAN [25]	72.0
Second-order Temporal Pooling [48]	72.4
HOK + second-order + IDT-FV [49]	73.3
Chained multi-stream networks [50]	76.1
**STA-TSN (RGB + Flow)**	**78.4**

### Spatial-temporal attention visualization

**Fig 6:**
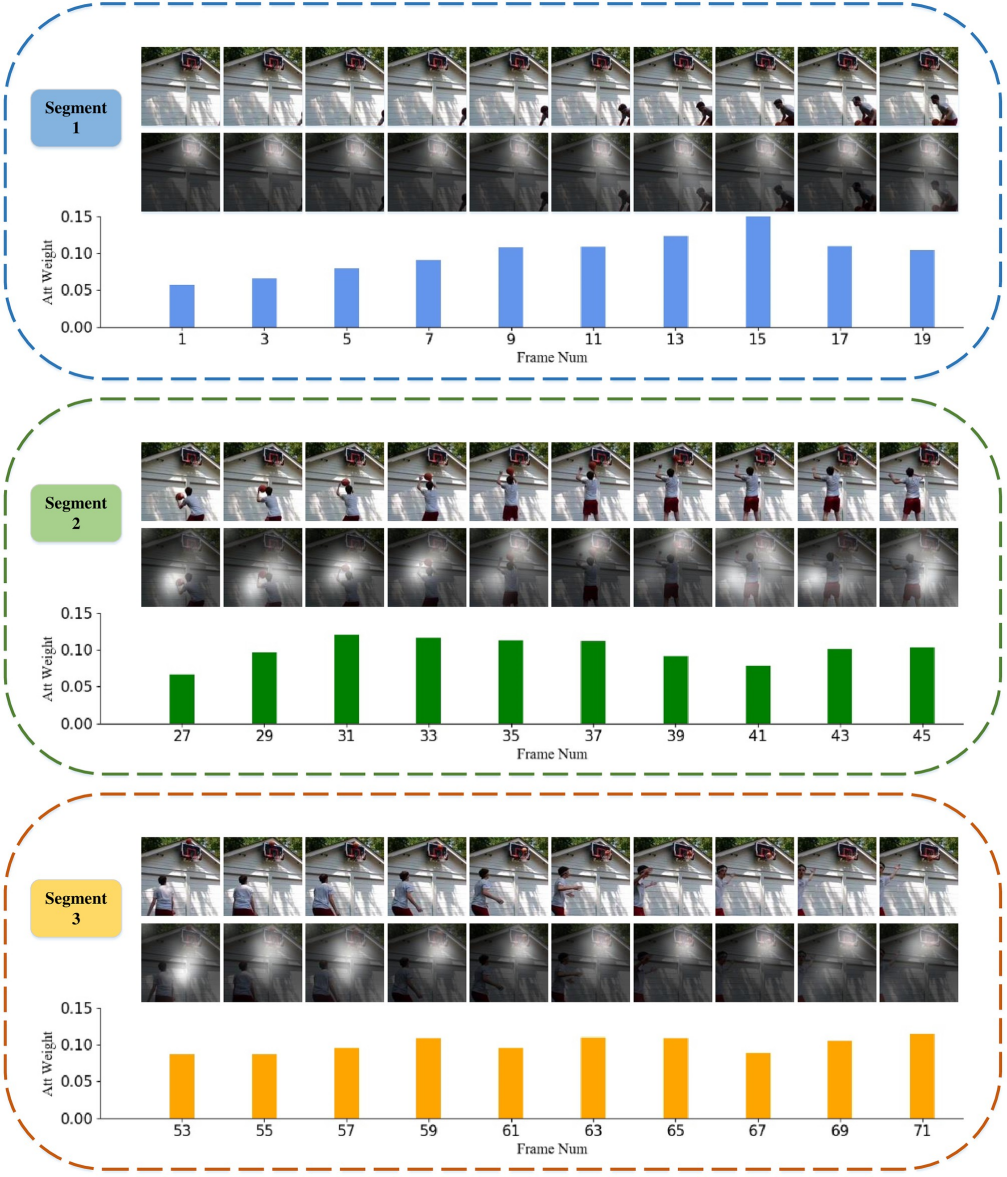
The visualization results of our STA-TSN for “shoot ball” in HMDB51. The first line is RGB images cropped from the center to a size of 224×224. The second line is RGB images with spatial attention masks, where the brightness indicates the focus level in space. The third line is the histogram of the temporal attention weights of the corresponding frames. Reprinted from [10] under a CC BY license, with permission from IEEE publisher, original copyright 2018.

As shown in Fig 6, we visualize the spatial and temporal attention results obtained by our STA-TSN using the action of “shoot ball” as an example. Specifically, each dashed box in the figure represents a segment of the video. The first line of each dashed box represents RGB images cropped from the center to a size of 224×224, and the second line is the spatial attention map for each frame obtained by fusing the original images and the spatial attention maps that have been upsampled to a size of 224×224, where the brightness of each area on the images indicates the strength of the spatial focus. The third line is the histogram of the temporal attention weights of the corresponding frames in each segment.

As can be seen from the images in the second line, our model focuses more on the basket area when the human does not make a shooting action. When the human starts to make the shooting action, our model shifts its attention to the human’s shooting posture. Therefore, our module accurately locates the spatially focused area in the image.

In terms of temporal attention, in segment 1, the temporal attention weight generally rises with the appearance of the human. In segment 2, frames 29–37 are the core part of the entire shooting action. During this period, our model also has higher attention weights. Since the shooting action is completed in segment 3, the temporal attention weights of the frames in this segment tend to be stable. Therefore, our model effectively realizes the exploration of key frames in the TSN.

## Conclusion

In this paper, we propose a spatial-temporal attention temporal segment network (STA-TSN) for action recognition in videos, which preserves the ability of TSN to capture long-term information and achieves adaptive focus on spatio-temporal key features. First, we employ a multi-scale spatial focus feature enhancement strategy to obtain the global feature representation with spatial attention rather than using GAP only in typical CNNs. Second, in order to give the TSN the ability to discriminate key frames, we develop an LSTM-based soft attention mechanism, which utilizes the temporal dynamic features explored by the LSTM to realize each key frames exploration in a segment. Besides, we design a temporal-attention regularization to guide our module to better explore key frames. Finally, we evaluate our model on four public datasets: UCF101, HMDB51, JHMDB and THUMOS14. The results show that our STA-TSN is better than baselines and outperforms other CNN-based, LSTM-based, and attention-based approaches on UCF101 and HMDB51, and also achieves better performance than other state-of-the-art methods. There are also some limitations in our current works, such as we only use RGB and optical flow modalities and just validate our module on the RestNet. In the future, we will first extend our model to more modalities and explore a more advanced fusion strategy across all modalities instead of average pooling. Second, we will try to transfer our module to more CNNs for action recognition.

## Supporting information

S1 File (ZIP)

S1 Data (PDF)
