# Supplementary material for: STA-TSN: Spatial-Temporal Attention Temporal Segment Network for action recognition in video
Source: PLoS One. 2022 Mar 17;17(3):e0265115. doi: 10.1371/journal.pone.0265115 (PMC8929560; doi:10.1371/journal.pone.0265115)
Supplement: S1 Data — (PDF) [file pone.0265115.s002.pdf]

UCF101：https://figshare.com/articles/dataset/UCF101_dataset/19179269
HMDB51：https://figshare.com/articles/dataset/hmdb5/19180622
JHMDB：https://figshare.com/articles/dataset/JHMDB/19179260
THUMOS14：https://figshare.com/articles/dataset/THUMOS14/19179266
